# Patient involvement in basic rheumatology research at Nijmegen: a three year’s responsive evaluation of added value, pitfalls and conditions for success

**DOI:** 10.1186/s41927-022-00296-6

**Published:** 2022-10-07

**Authors:** Maarten P. T. de Wit, M. I. Koenders, Y. Neijland, F. H. J. van den Hoogen, P. M. van der Kraan, F. A. J. van de Loo, H. Berkers, M. Lieon, A. van Caam, C. van den Ende

**Affiliations:** 1 Stichting Tools, Amsterdam, Netherlands; 2 Experimental Rheumatology, Radboud Institute for Molecular Life Sciences, Radboud University Medical Center, Nijmegen, Netherlands; 3 Department of Rheumatology, Sint Maartenskliniek, Nijmegen, Netherlands; 4 Department of Rheumatic Diseases, Radboud University Medical Center, Nijmegen, Netherlands; 5 STAP Panel, Nijmegen, Netherlands

**Keywords:** Basic research, Patient involvement, Patient research partner, Preclinical research, Responsive evaluation, Rheumatology

## Abstract

**Background:**

Empirical evidence for effective patient-researcher collaboration in basic research is lacking. This study aims to explore good working models and impact of patient involvement in basic rheumatology research and to identify barriers and facilitators.

**Method:**

A responsive evaluation of a three years’ participatory research project in a basic and translational laboratory research setting. Several working models for patient involvement were piloted and adapted if considered necessary. The study comprised surveys, interviews, training days, meeting reports, Q-sort exercises and field notes, and regular reflective team sessions with participant involvement. A qualitative analysis using thematic coding focused on impact, barriers and facilitators.

**Results:**

Thirteen patient research partners (PRPs) and fifteen basic researchers participated. PRPs experienced basic research as fascinating though complex to understand. Their initial role was mostly listening and asking questions. After several meetings equal and more meaningful relationships emerged. Researchers’ motivation increased by listening to patient stories. They learned about disease impact on daily life and to speak in understandable language. This enabled PRPs to learn about research and the pathogenesis of their disease. It inspired them to stay involved over a longer period. After three years, both parties preferred 1:1 contacts over collaboration in team meetings. A common language and respectful communication were important facilitators. Limitations were the complexity of disease processes for patients and the time commitment for researchers. Impact was reported as a sincere dialogue with multiple advantages for patients and researchers, and to a lesser extent than expected on the research process and outcomes.

**Conclusion:**

Patient involvement contributes to motivating young scientists in performing basic research projects. Patients and researchers valued the benefits of long-term one-on-one collaboration. These benefits outweigh the lack of direct impact on basic research goals and performance.

A plain language summary of the abstract is available (as) online Additional file 1.

**Supplementary Information:**

The online version contains supplementary material available at 10.1186/s41927-022-00296-6.

## Background

Patient and public involvement (PPI) in basic research is under-examined, especially when compared to clinical and psychosocial research [1, 2]. Existing challenges such as institutional barriers may play an important role. Laboratory-based research is traditionally not a patient-facing discipline, which may explain the lack of supportive guidance and opportunities for funding in this area [3]. Also personal concerns may prompt researchers’ reluctancy to engage patients in their projects. Early career researchers may refrain from engaging patients because of ethical issues such as how to define and identify the right patient, how to acknowledge and compensate patients for their input, and how to avoid tokenism? [4]. Some belief that PPI is time-consuming and may dilute their research objectives or even jeopardize ‘long-term value for short-term gains’ [3]. Finally, researchers may fear a lack of communication skills, public disengagement and the burden of expectation [3]. For patients, the technical language is challenging [5] as well as the expectation of having little to contribute [6]. For all of these reasons, evidence-based recommendations or best practices are still lacking. Some guides are available, but these are based on expert opinions [7, 8] rather than empirical evidence. The same holds true for documents on stakeholder-pharma collaboration, but also these are often based on consensus [9]. Although patient involvement has a long tradition [10, 11], it is only since the last decade that more empirical studies have been published in the field of rheumatology, focusing on building collaborative relationships in the context of laboratory-based pre-clinical research [5, 12–14].

In general it is thought that patients could and should play a role in basic research. A recent scoping review concluded that the most reported benefit is ‘providing a mutual learning opportunity’ and recommended more research on the impacts of patient engagement in preclinical laboratory research [15]. However, not much is known about how meaningful participation should be defined in this context and what conditions should be fulfilled. Meaningful does not refer to maximum involvement or to transforming power balances, but to opportunities for equal and respectful dialogue between patients and researchers that both parties experience as valuable [16]. We define patient participation as the active involvement of people with first-hand experience of a health condition in the design, conduct and dissemination of research. This approach is based on the ethical imperative to provide patients a say in research [4] and the assumption that the experiential knowledge of their disease and the health care system complements the evidence-based knowledge of researchers [5]. Whether patient participation is valuable in terms of impact is dependent on the assessment of benefits and caveats by all stakeholders involved.

To explore opportunities for patient involvement in basic research,^1^ the department of rheumatic diseases of the Radboud University Medical Centre (Radboudumc, Nijmegen, the Netherlands) initiated a pilot in the context of STAP (‘Key To Active Participation’), an initiative to set up a hospital-based patient panel to support the rheumatology research (both clinical and basic) at Radboudumc and Sint Maartenskliniek. The STAP panel comprises 41 patient research partners (PRPs) and meets twice a year. Members receive regular training and support, and provide the patients’ perspective in research projects. Here we report the results of the pilot and its follow-up to bring researchers and patients together in the research laboratory. Different formats for collaboration were chosen and evaluated with active engagement of all participants. Evaluation aimed to answer the following three research questions: What impact of patient participation in laboratory research is experienced or observed by the participants? What are factors that are supportive for constructive engagement of patients in a laboratory setting? What are perceived obstacles? As far as we know, this is the first case study that explored patient-researcher collaboration in basic rheumatology research.

## Methods

**Fig. 1:**
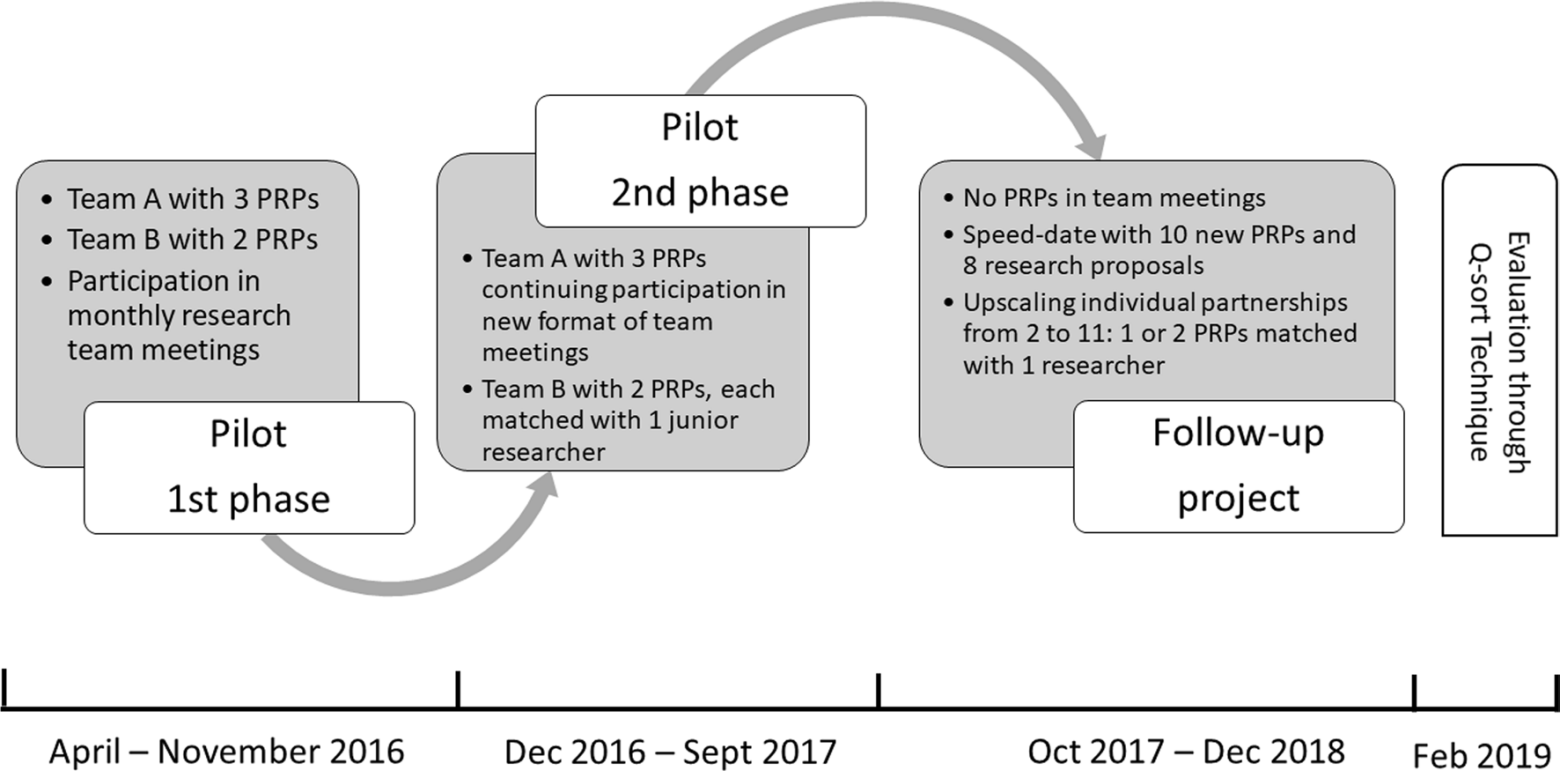
Project time line

A methodology of responsive evaluation [17, 18] was chosen as the most appropriate approach to summarize, analyze and report the results of a three years project to explore opportunities for patient involvement in basic rheumatology research (Fig. 1). This highly iterative approach centers on the expectations, perceptions and experiences of all participants and allows the analysis of changes over time. Responsive evaluation is in particular suitable to evaluate projects with an emergent research design, using a variety of qualitative and quantitative methods, and relies on contributions from all stakeholders along the way [19].

### Research team

The research team comprised the project coordinator (a specialized rheumatology nurse; YN), a senior health researcher (CvdE), three research team leaders from the lab (PK, FL, MK) and two patient research partners (HB, ML). The project coordinator guided the pilot, organized events and data collection, attended research team meetings and kept a comprehensive log book. She also conducted all interviews. Participating PRPs and researchers were actively involved in the design and conduct of the pilot and its evaluation, depending on interests and availability. They decided on the choice of format for collaboration, provided input in the development of interview protocols and survey forms, programs for training days and participated in evaluation and team meetings. As external advisor right from the start and responsible for the training of STAP panel members and researchers, MdW, a person with a rheumatic condition and a PhD in collaborative research, coordinated the Q-sort exercises and data analysis.

### Setting and participants

The project developed in three phases (Fig. 1). At the start of the first phase (Apr-Nov 2016) five PRPs of the STAP panel and two out of four research team leaders of the Laboratory of Experimental Rheumatology of the Radboudumc, showed interest to participate in the pilot. They decided that PRPs would join the monthly research team meetings, three PRPs in team A (focus: molecular processes in the context of drug development) and two PRPs in team B (focus: pathophysiology of osteoarthritis and scleroderma). None of the researchers had any experience with patient engagement. One researcher justified this approach as follows: “We try to apply knowledge that we jointly acquire along the way, in the context of our research projects; If this format of participation does not work, we adjust the format”.

At the first meeting of both teams it became clear that expectations needed to be tempered. It was agreed that participants would start, evaluate regularly and that the pilot could be stopped if participants did not see any benefits in continuation. During subsequent meetings one junior researcher presented a summary of ongoing work at a time. There was extensive room for PRPs to ask questions, followed by discussion.

During the second phase of the pilot (Dec 2016–Sep 2017) team A decided to continue participation in monthly meetings with another format: As preparation, the PRPs received a Dutch lay summary with three questions for the PRPs, formulated by the presenting researcher prior to the meeting. Team B decided that both PRPs would each join a single study on their own disease. They met with their researcher every six weeks to discuss results or to talk about disease-related topics. On request, the PRPs and researchers received customized training facilitated by the external advisor.

After evaluation of the pilot (Oct 2017) phase three started with a speed-date event (March 2018) with the purpose to replace participation in regular team meetings by nine new partnerships in couples of one PRP and one junior researcher (Fig. 1). A final training and evaluation day took place at the end of the project. Characteristics of all PRPs and researchers are provided in Table 1.

**Table 1:** Characteristics of participants in 1st and 2nd Phase of pilot and follow-up project

Researchers (n = 11)		Patient research partners (n = 8)	
Age (range)	33 (24–59)	60 (50–73)	Age
*M/F*	5/6	2/6	*M/F*
*Country of birth*			*Country of birth*
*Netherlands	11	7	*Netherlands
		1	*Suriname
*Education*			*Education*
* Lower education			*Lower education
* Middle education		3	*Middle education
* Higher education		4	*Higher education
* Academic education	7	1	*Academic education
* Post-academic education	4		*Post-academic education
*Job title*			*Diagnosis*^a^
* Team leader/(Ass) Prof	2	5	*Rheumatoid Arthritis
* Post-doctoral researcher	2	1	*Systemic Sclerosis
* PhD student	7	2	*Osteoarthritis
		1	*MCTD
*Years in research (range)*	10.1 (2–35)	20 (3–59)	*Disease duration years (range)*
*Experience with PPI*	None		

Ass. = Associate; MCTD = Mixed Connective Tissue Disorder; PPI = Patient and Public Involvement

^a^More than one diagnosis possible

### Data collection

Data were collected through meeting reports, surveys, interviews, training sessions, observations and field notes (logbook). Researchers and PRPs were interviewed by the coordinator (YN) before the start of the pilot and at the end of the first phase of the pilot. Reports of regular reflection meetings with all participants were made (YN) and PRPs were asked to fill in evaluation forms after each team meeting. Member check took place through circulation of each report with the opportunity for participants to comment. Two plenary meetings after three years used small group assignments and a Q-sort technique for final evaluation of all participants’ experiences. An overview of documents used for analysis is provided as Additional file 2.

### Data analysis

To ensure that findings were grounded in participants’ experiences, data were objected to inductive thematic analysis (YN, MdW) [20, 21]. Analysis focused on all types of impact and perceived barriers and facilitators for patient involvement. Documents were read and reread before allocating descriptive labels (codes) to identified units of meaning (sentences; quotations) [22]. Characteristic concepts of impact were formulated as statements and included in two Q-sort assignments for a final evaluation by the participants. The first asked participants to sort statements individually according to the question “What is your personal experience regarding the impact of patient involvement in basic research?”. The second assignment asked participants to sort statements reflecting similar concepts in separate piles. Then they gave a name to each pile clarifying what these statements have in common. Main categories of impact, facilitators and barriers were derived from the outcomes of the Q-sort exercises and previous data analyses and, after discussion, agreed by the research team.

### Quality measures

We followed the GRIPP-2 checklist for reporting patient involvement in scientific research (Additional file 3) [23]. Participating researchers and PRPs provided written informed consent. Regular member checks and the approach of responsive evaluation prevented the loss of meaningful input during the stages of analysis, interpretation and reporting. Triangulation, comparing data from different sources of evidence, helped to look for confirmative or inconsistent findings.

## Results

**Fig. 2:**
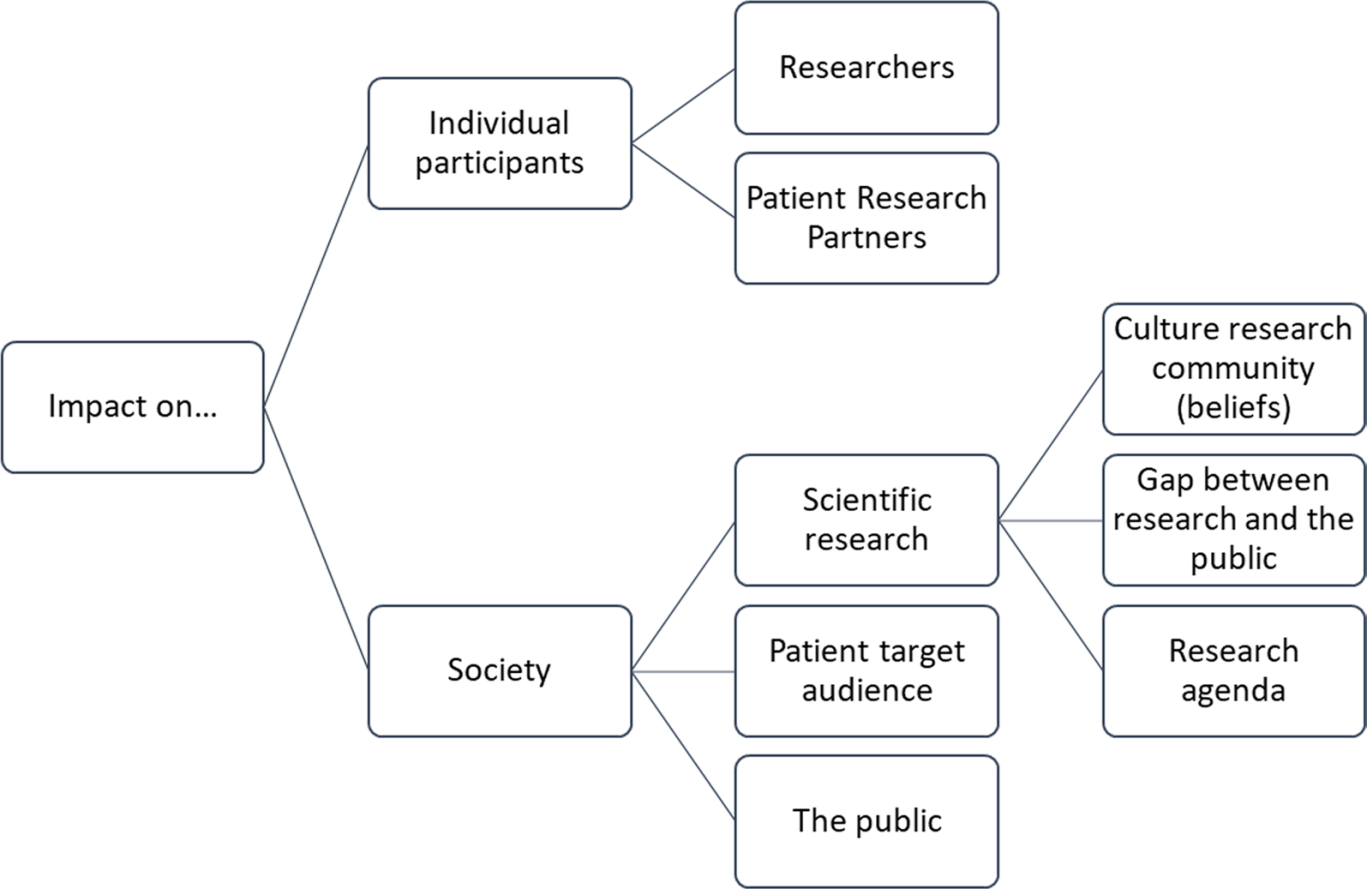
Main impact categories from the data analysis

During the first phase of the pilot, data were collected (Additional file 2) through eleven meeting reports, 23 interview reports and seventeen evaluation forms. During the second phase of the pilot, data were collected through eleven meeting reports, six interview reports and eight evaluation forms. During the follow-up project, data were collected through three meeting reports and 33 individual Q-sort assignments. After analysis of all resources, the team distinguished two main categories, personal and societal impact, and five subcategories: impact on the researcher, the PRP, the research process, the target patient audience and the public (Table 2, Fig. 2).

**Table 2:** Overview impact of patient involvement in basic research

Personal impact		Societal impact	
Researchers	PRPs	Research process	Patient target audience and general public
*Holistic view* Attaining a more holistic view of people with a rheumatic condition: Patients get a face Changing perceptions as the result of learning disease impact on daily life *Learning to explain research in lay language* Better lay summaries in grant applications Better presentations for lay audiences *Prompted to consider clinical relevance of research* Getting detached from own research (helicopter view) and encouraged to look at research from a patient perspective Better understanding of the societal impact of research	*Better understanding of basic research* Research comes to life To see the bigger picture More realistic expectations: Understanding why research sometimes fails and why progress takes often a long time *Better understanding own disease* Understanding the importance of pathophysiological processes for explaining disease activity and for potential treatment options *Personal satisfaction* Sharing personal experience with fellow PRPs and researchers Receiving recognition	*Legitimacy* Broader stakeholder representation Patients’ voices are heard PRPs become sparring partner *Research agenda* Reality check: is research studying questions that are important to patients? Identifying new research topics such as: Pathophysiological factors of fatigue (research proposal submitted) Side effects of MTX (no follow-up) *Recruitment* PRPs reviewing patient information letters and other patient materials [*not seen in this study*] *Reduced gap between research and the public* *Motivation* Enhanced motivation as result of better understanding of the clinical burden of disease	*Improving health care* Better patient information as the result of PRPs reviewing lay summaries PRPs and researchers giving presentations for scientific and patient audiences *Dissemination* PRPs giving interviews for media Combined presentations on national symposia as well as internal educational meetings

MTX = Methotrexate; PRPs = Patient Research Partner

## Personal impact

### Impact on the researcher

Prior to the pilot, some researchers had doubts about the added value of PRPs: “I am not doing research for patients, but because I am curious to know what is happening in the cells.” Other researchers were excited when they heard that patients would attend their team meetings and believed that PRPs would provide meaningful contributions to the design and conduct of their research.

*Motivation—*Researchers became more motivated by talking with patients, sometimes meeting a patient for the first time. They made serious efforts to speak in understandable language which was experienced as a useful exercise, particularly in the light of the increasing demands from funding agencies for increased engagement with patients, and the involvement of patients in the assessment process:

As researcher in the lab you normally never speak with patients. Now you feel empowered, knowing why you do this research (…) With his lay questions my PRP forces me to explain with simple words what I am doing. This is very beneficial when I am writing my grant proposals.

During the second phase of the pilot, one of the senior researchers concluded that junior researchers obtained “a more holistic view”: “They now have the chance to do research with patients and not only on patients”. Over time ‘talking to’ changed into ‘talking with’, and perceptions of researchers changed as the result of more insight in challenges of daily living that patients encounter. This was confirmed by one of the junior researchers who said: “I can see now for whom I am doing my research. In the past, actually I only did it to be able to write my thesis”.

A senior researcher introduced the challenge of translating the most important clinical problems in osteoarthritis into goals for basic research in the EULAR study group for osteoarthritis. It became clear that the perspective of patients differs from that of researchers. For the latter, osteoarthritis is characterized by abnormalities in structure and function (genes, proteins and cells, signaling, and metabolic pathways), while for patients the condition is a collection of symptoms: pain, functional limitations, aesthetic damage and loss of daily and social activities. Researchers and PRPs became coauthors of this manuscript [24].

Table 3 offers a list of ten prioritized impact statements for researchers compared to those of PRPs derived from the Q-sort exercise.

**Table 3:**
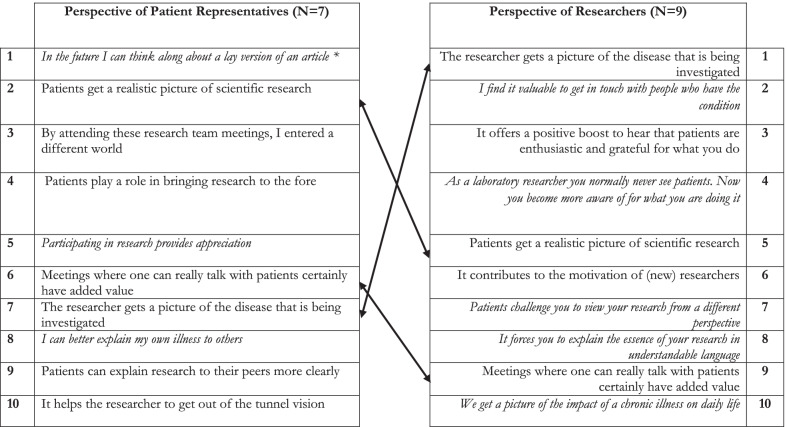
Most reported impact by PRPs and researchers—results from the Q methodology

*Italic represents statements that were only asked to one of the two groups

### Impact on the patient research partner

PRPs experienced basic research as complex and difficult to comprehend, mostly because of the language used by researchers. Their degree of participation was therefore limited, and although the interaction mainly consisted of listening and asking questions for clarification, it was reported worthwhile because researchers were willing to present their research in lay language and explaining terms like ‘significant’ and ‘biomarkers’. It motivated PRPs to stay involved and enabled them to see the bigger picture of basic research. They started to understand why research takes such a long time and actually never ends, and how research is funded and priority-setting is necessary. They also learned more about their own disease.

### Societal impact

#### Impact on research process

##### Research agenda

Participants started collaboration by attending monthly team meetings. After a while the participants agreed that the input of PRPs during these meetings was limited and time consuming, with the exception of a few cases where researchers were triggered by patient stories to take research questions into the lab. During an informal gathering a researcher reported that by talking with patients he became intrigued by the topic of fatigue: “In our laboratory we never paid attention to this phenomenon”. A patient research partner said:

Researchers really listen to our needs. On our request, for example, a research proposal has been submitted to do research on fatigue in scleroderma because we all suffer from this.

In another meeting researchers were surprised to hear several patient stories about side-effects of methotrexate and expressed interest to put this topic on the research agenda. One researcher acknowledged that the insights into the disease process of his PRP influenced some of his research decisions.

##### Bridging the gap

Patient research partners are able to bridge the gap between research and the general public. They supported effective recruitment of study participants in lab-based research projects, and dissemination of research findings to patient audiences and the public. In this pilot project PRPs helped in writing lay summaries of research projects and reviewing consent forms and procedures. During the follow-up phase, several PRPs reported involvement in the development of educational materials for patients and received invitations, sometimes with researchers, to give presentations for patient organizations and for the academic community.

##### Motivation

Researchers reported appreciation of the interaction with patients, mostly during informal moments such as coffee breaks or before and after the official team meetings. As already mentioned, they developed the skills to explain the goals and importance of their research in understandable language and enjoyed giving presentations for the patient panel as well as for a wider patient audience.

#### Impact on the target audience and the public

It is assumed that patient involvement in research leads to improved health care as a result of greater emphasis on patient relevant outcomes and more focus on the feasibility and practical use of new health interventions. However, participants agreed that it is unrealistic for a three years project to expect a direct impact of patient involvement on health outcomes for patients.

### Facilitators

Developing meaningful and sustainable partnerships in laboratory research takes time. After three months the atmosphere at the monthly team meetings became open and relaxed, and more equal relationships emerged. Time to develop realistic expectations of patient involvement and a common language, and respectful communication were key success factors (Table 4).

**Table 4:** Facilitators and barriers for successful patient participation in laboratory research

Facilitators	Barriers
Management of expectations: allowing time to develop realistic expectations Education of principles of patient-researcher collaboration Development of a common language Respectful communication, also during informal breaks Professional support by the coordinator Support by the leadership Enabling 1:1 contact Speed date for ensuring effective researcher-PRP partnerships Adequate reporting and storage of patient and public summaries and information	Complexity of pathogenetic processes for patients Time commitment for researchers Formal setting of monthly team meetings Planning meetings is challenging, due to PRP characteristics (e.g. morning stiffness) Frequency and intensity of 1:1 interactions are vulnerable because of the disease of the PRP

*PRP* patient research partner

The coordinator played a crucial role by facilitating communication, ensuring continuity and organizing education of PRPs and researchers on demand. Training was not focused on biology or scientific research, but on principles of collaborative research, including role play of a first introduction meeting of a PRP and researcher.

At the end of the pilot, participants almost unanimously preferred 1:1 contacts over patient involvement in team meetings. In particular the opportunity for more informal conversations was reported favorable for the exchange of personal information and experiences. Researchers motivated their preference as follows: “If you agree on regular appointments, the PRP grows automatically with the research” and “Because you have to explain a lot, it is easier and more efficient to talk 1:1”. PRPs clarified their wish for 1:1 contacts: “The contact is very authentic” and “It is all so complex, I like to focus on just one study”.

When considering a PRP recruitment strategy, inviting patients to apply for the role as PRP worked better than physicians selecting patients. People who applied for a role as PRP had a strong intrinsic motivation, were curious and held an interest in basic research. Using speed dating for matching patients and researchers turned out to be a good strategy for building promising partnerships.

Not only the coordinator’s support, also the support from the leadership turned out to be a strong facilitator. One of the participating senior researchers acknowledged in a public inaugural lecture:

Since the pilot we try to involve patients as intense as possible in our research. With the steps we have already made, it is apparent that this close interaction between researcher and patient has a clear added value.

Direct support by the researchers comprised distribution of pre-meeting reading material and Dutch lay summaries. “You have to prepare your presentations differently and invest in slides with simpler layout”, as one PhD student explained.

Based on these facilitators we developed a step-by-step approach for establishing sustainable partnerships between patients and researchers in the laboratory (Additional file 4).

### Barriers

Barriers were the complexity of molecular biology for patients and the time commitment for researchers. Preclinical research is complex and pathogenetic processes are difficult to explain to lay people. The initial format of team meetings and researchers presenting their project formed a good start, but did not encourage much interaction. There was a need to follow-up with more opportunities for informal and in-depth communication and dialogue. The format of individual partnerships became the preferred option.

Matching of PRPs and researchers did not always work out well. PRPs have their own interests and not every study is of equal importance to all PRPs. For this reason the speed date was introduced and became an effective way to ensure a natural fit between researchers and PRPs. During the project two speed date meetings were organized, resulting in fourteen new partnerships, including for the first time three clinical projects. Some postdocs and last year PhD students wrote long-term research proposals and formulated, together with their PRP, common objectives and a strategy for dissemination of findings. Relationships were sustained, demonstrating that researchers were motivated to continue collaboration with PRPs in new or follow-up studies. It enabled them to incorporate PRPs input right from the conception of their project.

Finally, the time commitment was often mentioned by young researchers: “It takes a long time to introduce a PRP to the project and to make sure that they can think and talk about the content as equal partners”. However, despite this initially reported barrier, participating researchers acknowledged that their time investment was not in vain because PRPs that continue their involvement at the department will bring their acquired knowledge and expertise to other projects.

## Discussion

This three years’ study demonstrates that the perception of the value of patient engagement in laboratory research can change as the result of personal relationships with patients. The original thought of young researchers and other team members “I am doing research because I am interested in molecules” gradually changed into a genuine interest in the life and perspectives of people with the condition under research. Although they initially felt that patients lack knowledge of molecular biology, perceived as necessary to provide a meaningful contribution, they learned, accepted, and appreciated that the added value of PRPs comprised something different. In the end, all participants found the collaboration worthwhile. Impact was reported as a diversity of benefits for patients, researchers, the research process and society, but little added value was seen on the goals and content of basic research, except for the addition of patient relevant topics to the research agenda. These findings are confirmed by publications from the FP7 multi-disciplinary translational research project “EuroTEAM” (Towards Early biomarkers in Arthritis Management). There, many PRPs reported a language gap between patients and researchers, and perceived the opportunities for involvement in preclinical research limited [5]. The authors confirmed that the role and impact of PRPs in their work packages on psychosocial research were more tangible and obvious than in laboratory-based work packages. However, researchers involved in several work packages, learned to recognize and accommodate opportunities for patient involvement in basic research projects [13].

EuroTEAM participants, both PRPs and researchers, reported many similar benefits of patient involvement as we identified. For instance, PRPs reporting access to the latest research findings and the opportunity to give something back for care received. Researchers found it very rewarding to work with PRPs, their perceptions of patient involvement had evolved in a positive direction and they saw a greater input from PRPs in their future projects.

The substantial role of patients in advancing dissemination (developing understandable patient information, motivating junior researchers, informing patients) was emphasized [5, 13]. These findings are also consistent with a systematic review that showed that the success of PPI relies on the processes of engagement and that the impact of PPI on the people involved is of greater value than on the research [25]. A question that we could not answer is whether this is only true for young-career scientists or also for senior researchers. Another study found a very similar impact on researchers involved in Parkinson disease [26]. They reported gaining new knowledge by talking with patients with the condition. This resulted in a better understanding of the issues that really matter to them. Having face-to-face contacts changed their professional values and created a sense of the ‘people’ behind the data. Finally, like in our and many other studies, the researchers gained new skills in communicating with the public. Elsewhere, four PhD students, sharing their experiences with different approaches of PPI, did face some challenges (ensuring funding for PPI and developing group work skills), although they reported a positive effect on each study progression and an improvement in their self-esteem. It also helped them to feel less isolated as doctoral researchers [27].

Important facilitators for the change in perceptions of all participants were, apart from the extensive support by the project coordinator, the long duration and the initial small size of the pilot, the involvement of immediate stakeholders in decisions regarding the project design, the exchange of realistic, mutual expectations, and regular interim evaluations and subsequent reformulation of objectives. Also starting with only researchers who were intrinsically motivated to experiment with different formats of patient participation led to positive experiences that inspired newcomers. Training sessions started originally as separate events for PRPs and researchers, but participants rapidly decided that they preferred joint workshops. PPI training of researchers is often recommended, although our experiences may imply that the value is overestimated. Training provided top-down and designed by researchers is less productive than providing guidance and coaching on request of any stakeholder and developed in close collaboration with all involved [28]. Finally, the support of the leadership turned out to be a key facilitator. The project was developed bottom-up without a steering group imposing strategies or decisions on the participants. As an example, organizing a speed date meeting to match new research projects (and researchers) with PRPs was a suggestion of one of the participants that was well received by others.

We have learned that patient participation depends highly on the establishment of long term relationships and tailored formats of support and education. The latter are not focused on ‘becoming a researcher’ (proto-professionalisation) [29], but on avoiding risks of tokenism by ensuring meaningful formats of involvement. Our findings confirm the assumption that meaningful involvement of patients—meaning: to be involved in the design, conduct and dissemination of a study—can only emerge when mutual respect, a supportive environment and equal relationships have been carefully established [14].

This study is based on the dialogue model, as developed by Tineke Abma c.s. [30]. According to this theory, patient participation is characterized by mutual learning and creating greater understanding of the perspective of other stakeholders rather than a simply transfer of power [19, 31]. In this regard the often used ladder of participation with increasing levels of influence on the research process is less adequate to study involvement in laboratory research.

We found that PRPs and young researchers preferred the 1:1 partnership which might be seen as incongruent to the EULAR recommendation to have at least two PRPs on a research team [8]. However, a recent pilot in Birmingham, the Student Patient Alliance (SPA), followed this same format. Rheumatology PhD students were put in contact with one or more patient research partners with the aim to informally facilitate patient involvement in their research project and to develop their skills in working and communicating with members of the public. The pilot became a success. Both students and PRPs were enthusiastic and in 2020 the pilot was extended to include all new rheumatology PhD students [32].

Strength of this study are the duration (three years) and the approach of responsive evaluation, a formative method to demonstrate the impact of a phenomenon from the perspective of the directly involved stakeholders. The participants, both researchers and PRPs, were actively involved in the design of the study, the data collection and interpretation. This created a strong feeling of ownership over the project. The help of a qualified coordinator in collecting data, matching researchers and PRPs ensured the continuity of the research. The use of mixed methods enabled triangulation of findings which increased the transferability of our results.

Representation of the patient perspective, like many studies evaluating PPI, is also a limitation in this study (Table 1). The PRPs had a more than average educational level and most were white, female and of higher age. It remains difficult to address the issue of equity and diversity which may require radical other approaches of involvement and recruitment. Another limitation is the fact that we demonstrated the impact of PPI in basic research through a combination of qualitative methods. Further research should quantify the benefits of PPI in basic research, for instance to what extend PRPs contribute to creating more supportive conditions for basic research. Does PPI result in better legislation and less bureaucracy, more research funding, improved recruitment and retention rates, and ultimately better outcomes for patients?

Although the added value of patient involvement seems less obvious in basic research than in clinical research, the potential advantages should not be underestimated. This study shows that researchers and PRPs are positive about the opportunities for meaningful involvement of PRPs in basic research when these opportunities are developed in partnership.

## Conclusion

This three years’ study demonstrates that the perception of the value of patient engagement in laboratory research can change as the result of personal relationships with patients. Although the impact of PPI on pre-clinical laboratory research is limited, all participants involved reported substantial benefits. Rather than a change of power balances, collaboration leads to an increased understanding of other stakeholders. Researchers and patient research partners appreciated the long-term one-on-one relationship above regular team meetings.

## Supplementary Information

**Additional file 1.** Plain language summary.

**Additional file 2.** Overview of documents included in the thematic analysis.

**Additional file 3.** GRIPP2 checklist long form.

**Additional file 4.** Road map for patient involvement in basic research (step-by-step plan).

## Data Availability

Due to the nature of the consent, reports, transcripts and any information that could identify participants is not available. Further information is available by application to the corresponding author.

## Abbreviations

EULAR: European Alliance of Associations for Rheumatology
EUROTEAM: Towards Early biomarkers in Arthritis management
FP7: Seventh framework programme of the European Community
GRIPP2: Checklist for reporting of patient and public involvement in health and social care Research
PhD: Doctoral study
PPI: Patient and Public Involvement
PRP: Patient Research Partner
STAP: Key to Active Participation

## Footnotes

1 In this article the term ‘basic research’ is used as a synonym for pre-clinical or laboratory research.
