# Supplementary material for: Patient involvement in basic rheumatology research at Nijmegen: a three year’s responsive evaluation of added value, pitfalls and conditions for success
Source: BMC Rheumatol. 2022 Oct 7;6:66. doi: 10.1186/s41927-022-00296-6 (PMC9540713; doi:10.1186/s41927-022-00296-6)
Supplement: Supplementary file 1 — Additional file 1. Plain language summary. [file 41927_2022_296_MOESM1_ESM.pdf]

Patient involvement in basic rheumatology research at Nijmegen. A three year's responsive evaluation of added value, pitfalls and conditions for success.

M. de Wit, M.I. Koenders, Y. Neijland, F.H.J. van den Hoogen, P.M. van der Kraan, F.A.J. van de Loo, H. Berkers, M. Lieon, A. van Caam, C. van den Ende

Plain language summary

Objective: Evidence for effective patient-researcher collaboration in basic or laboratory research is lacking. Our aim was to study the impact of patient involvement in basic rheumatology research and to answer the following research questions: 1. How can researchers and patient representatives work together? 2. What supports successful partnerships? And 3. what hampers successful partnerships?

Method: During a 3 years' research project, we evaluated the collaboration between researchers and patient representatives. This was done through surveys, interviews, training days, meeting reports, card-sorting exercises and making field notes. As a team we gathered regularly to discuss the progress of the project. By reading and analysing all kinds of documents we tried to answer our research questions.

Results: In total 13 patient research partners (PRPs) and 15 basic researchers participated. PRPs experienced basic or laboratory research as complex because of the used scientific language. Their initial role was mostly listening and asking questions. After several meetings the atmosphere relaxed and equal relationships emerged. Researchers' motivation to conduct research increased by talking regularly with PRPs. They learned to understand disease impact on daily life and to speak in understandable language. This enabled PRPs to learn about research and how their own disease starts and further develops. It inspired them to stay involved over a longer period of time. After 3 years, both parties preferred 1:1 contacts over collaboration in a team. Over time the number of partnerships increased. A common language and respectful communication was the most important factor that helped to create meaningful

partnerships. Barriers were the limited understanding of the complexity of biological processes for patients and the time commitment for researchers. All participants found the collaboration worthwhile. Impact was reported as a meaningful dialogue with personal benefits for patients and researchers. Impact of patient involvement on the research process and the outcomes of basic research was limited.

Conclusion: Patients and researchers experience valuable benefits from long-term one-on-one collaboration. These benefits outweigh the lack of direct impact on research processes and outcomes.
