# Supplementary material for: Patient involvement in basic rheumatology research at Nijmegen: a three year’s responsive evaluation of added value, pitfalls and conditions for success
Source: BMC Rheumatol. 2022 Oct 7;6:66. doi: 10.1186/s41927-022-00296-6 (PMC9540713; doi:10.1186/s41927-022-00296-6)
Supplement: Supplementary file 2 — Additional file 2. Overview of documents included in the thematic analysis. [file 41927_2022_296_MOESM2_ESM.pdf]

**Online supplement 1** Overview of documents included in thematic analysis (excluding Logbook components)

| Preparation phase | Meeting reports | | Interview reports RE | PRP | Survey forms RE | PRP | Q-sort assignments | |
|---|---|---|---|---|---|---|---|---|
| 1-10-2015 | (1) | | | | | | | |
| 27-10-2015 | (1) | | | | | | | |
| 16-11-2015 | (1) | | | | | | | |
| 12-1-2016 | (1) | | | | | | | |
| February | | | 6 | | | | | |
| **Phase 1** | **A** | **B** | **RE** | **PRP** | **RE** | **PRP** | | |
| March | 1 | 1 | | | | | | |
| April | 1 | 1 | 6 | | 1 | 3 | | |
| May | 1 | 1 | 6 | 5 | | 4 | | |
| June | 1 | 1 | | | 8 | 1 | | |
| July | 1 | 1 | | | | | | |
| **Subtotal** | **5** | **5** | **18** | **5** | **9** | **8** | | |
| June Steering group | 1 | | | | | | | |
| **Phase 2** | **A** | **B** | **RE** | **PRP** | **RE** | **PRP** | | |
| October | 1 | 1 | | | 1 | 1 | | |
| November | 1 | 1 | | | | | | |
| December | 1 | | | | | | | |
| January '17 | 1 | | 1 | 1 | 1 | 2 | | |
| February | 1 | | | | 1 | 1 | | |
| March | 1 | | | | | | | |
| May | 1 | | | | 1 | | | |
| June | 1 | | | | | | | |
| June EULAR abstract | 1 | | | | | | | |
| September | | | 1 | 1 | | | | |
| October | | | 1 | 1 | | | | |
| **Subtotal** | **9** | **2** | **3** | **3** | **4** | **4** | | |
| **Follow up** | **A** | **B** | **RE** | **PRP** | **RE** | **PRP** | **RE** | **PV** |
| March 2018 Speed-date | 1 | | | | | | | |
| June SG | 1 | | | | | | | |
| October Training day | 1 | | | | | | | |
| Evaluation February '19 | | | | | | | 18 | 14 |
| **TOTAL** | **25** | | **29** | | **25** | | **32** | |

Legend: A (Research Group A); B (Research Group B); EULAR (European Alliance of Associations for Rheumatology); PRP (Patient Research Partner); RE (Researcher); SG (Steering Group meeting).
