# Supplementary material for: Patient involvement in basic rheumatology research at Nijmegen: a three year’s responsive evaluation of added value, pitfalls and conditions for success
Source: BMC Rheumatol. 2022 Oct 7;6:66. doi: 10.1186/s41927-022-00296-6 (PMC9540713; doi:10.1186/s41927-022-00296-6)
Supplement: Supplementary file 3 — Additional file 3. GRIPP2 checklist long form. [file 41927_2022_296_MOESM3_ESM.pdf]

Online Supplement 1

GRIPP2 checklist long form

Section 1: Abstract of paper

1a: Aim	To explore the impact of patient involvement in basic rheumatology research and to identify facilitators and barriers.
1b: Methods	A responsive evaluation of a 3 years' participatory research project, comprising surveys, interviews, training days, meeting reports and field notes, and regular reflective team sessions with participant involvement. A qualitative analysis using thematic coding focused on impact, barriers and facilitators.
1c: Results	The initial role of patients was mostly listening, sometimes asking questions. After three months the atmosphere became more relaxed and equal relationships emerged. Researchers' motivation increased by talking with PRs, sometimes for the first time. They learned to understand disease impact on daily life. They learned to speak in understandable language, which enabled PRs to learn more about research and their own disease. It inspired patients to stay involved over a longer period. After 3 years both parties preferred 1:1 contacts over collaboration in team meetings. Over time the number of partnerships increased significantly. All participants found the collaboration worthwhile. Impact was reported as many benefits for patients, researchers, research agenda and research process, but little on actual results of basic research.
1d: Conclusions	Both patients and researchers experience valuable benefits from long-term collaboration. They outweigh the lack of direct impact on research processes.
1e: Keywords	Among others: Patient Involvement; Patient Research Partner

Section 2: Background to paper

2a: Definition	Patient involvement in this study was defined as the involvement of people with personal experience of the condition under research (i.c. a rheumatic condition). In this article we use the term "Patient Research Partner" (PRP) which is used within rheumatology research groups such as EULAR, OMERACT and GRAPPA. In this form we use patients which can also mean PRP.
2b: Theoretical underpinnings	This study is based on the dialogue model, as developed by Tineke Abma c.s. According to this theory, patient participation is characterized by mutual learning and creating greater understanding of the perspective of other stakeholders rather than a simply transfer of power. In this

	regard the ladder of participation with increasing levels of influence on the research process is less adequate to study involvement in laboratory research. Abma, T.A. & J. Broerse (2010) Patient participation as dialogue: setting research agendas, *Health Expectations*, 13(2):160-73; Abma, T.A., C. Nierse & G.A.M. Widdershoven (2009) Patients as research partners in responsive research. Methodological notions for collaborations in research agenda setting, *Qualitative Health Research*, 19(3): 401-415.
2c: Concepts and theory Development	We used the dialogue model as elaborated by Abma c.s.

Section 3: Aims of paper

3: Aim	To report the results of a responsive evaluation of a 3 years' participatory research project in the area of basic rheumatology research

Section 4: Methods of paper

4a: Design	Patients were involved in the study through forms of consultation (surveys, interviews, training days) and direct collaboration (active participation in preparatory meetings, review of training programs and documents). Patients also joint steering group meetings and regular reflexive team sessions.
4b: People involved	In this study five patients participated in the pilot and 11 in the follow-up study. All had a rheumatic condition.
4c: Stages of involvement	Patients were involved in the conception of the project proposal. In the 1st phase of the pilot 5 patients participated in 6 monthly research team meetings. In the 2nd phase 3 patients continued participating in research team meetings while two patients were matched with two individual junior researchers. In the follow-up study all 5 patients stayed involved in the project.
4d: Level or nature of involvement	Patients entered the research teams as listeners. The level of their involvement gradually became more equal, despite the fact that one-way communication and limited interaction was reported.

Section 5: Capture or measurement of PPI impact

5a: Qualitative evidence of impact	Through surveys, interviews, training days, meeting reports, field notes and regular reflective team sessions, we collected evidence for the impact of patient involvement on the research process, the patient representative and the researcher.
5b: Quantitative evidence of impact	Due to the low number of participants we did not quantitatively assess the impact of patient involvement
5c: Robustness of measure	Responsive evaluation is a validated approach to assess the impact of a complex phenomenon as patient involvement. There are numerous publications on the use of

	responsive evaluation in participatory (health) research. The robustness is ensured by the intense and sustainable involvement of all participants during all phases of the research process.

Section 6: Economic assessment

6: Economic assessment	Because of the small scale of this pilot project, it is not possible to report economic impact data; also because of the different forms of participation that each have different financial consequences. However, costs of the project included: Salary of part-time coordinator, reimbursement of travel costs of patients, hours of researchers for the time they invested in meetings with patients and preparation, extra meetings with patients, and expenses of training and evaluation meetings.

Section 7: Study results

7a: Outcomes of PPI	The main focus of this manuscript is the evaluation of the impact of PPI in basic research. Personal and societal impact is distinguished (see Table 2). Table 3 provides an overview of priorities in impact according to Patient Representatives versus researchers.
7b: Impacts of PPI	We have not made a distinction between outcomes and impact. We used both concepts as synonyms for added value. However, we did describe some 'barriers' that might be perceived as '*negative'* outcomes of PPI, such as the time-consuming character of PPI.
7c: Context of PPI	We did not identify any contextual factor in this study
7d: Process of PPI	We have included a short summary of facilitators and barriers of PPI (see also Table 4).
7ei: Theory development	Our intention was to provide a case study of a pilot and follow-up project, evaluating the impact of PPI in basic research. It is still too early to draw conclusions regarding theory development.
7eii: Theory development	We did not test any theory concept or hypothesis
7f: Measurement	We followed an approach of responsive evaluation which did not include any *measurement outcomes*, but collected perceptions of impact according to the *participants*.
7g: Economic assessment	No quantifiable data were collected. See section 6.

Section 8: Discussion and conclusions

8a: Outcomes	In the two pilot phases as well as in the follow-up phase, decisions about the preferred format of involvement were in all cases the result of a shared decision making between the participating patient representatives, researchers and the project coordinator.
8b: Impacts	See 7a and 7b

8c: Definition	We looked at the role of patients as *patient representatives*, meaning participation in research teams as equal participants. This role has not been fully established. New follow-up projects should explore more extended opportunities for PRs to act as collaborative partners.
8d: Theoretical underpinnings	See 7e
8e: Context	This study focused in particular on the context of basic (laboratory) research. As such, it is an innovative study because no published empirical longitudinal evidence exists till now.
8f: Process	We followed an emergent design which facilitates maximum involvement and influence of all stakeholders (i.c. early career researchers and patients) on the process.
8g: Measurement and capture	We used an approach of responsive evaluation to collect empirical data on the impact of patient involvement in basic research. This resulted in a description of the perceived impact of PPI according to the two most involved stakeholders
8h: Economic assessment	This was a pilot study and no quantifiable data were collected. See section 6.
8i: Reflections/critical perspective	We have learned and experienced that an approach of responsive evaluation is a valid (reliable), feasible and effective method to demonstrate the impact of a phenomenon like patient involvement in research. By practicing what you preach (user involvement) – in our case by actively involving both researchers and PRs in the design of the study, we created a strong feeling of ownership over the project among participants.
