# Supplementary material for: Patient involvement in basic rheumatology research at Nijmegen: a three year’s responsive evaluation of added value, pitfalls and conditions for success
Source: BMC Rheumatol. 2022 Oct 7;6:66. doi: 10.1186/s41927-022-00296-6 (PMC9540713; doi:10.1186/s41927-022-00296-6)
Supplement: Supplementary file 4 — Additional file 4. Road map for patient involvement in basic research (step-by-step plan). [file 41927_2022_296_MOESM4_ESM.pdf]

Online supplementary file 4

Road map for patient involvement in basic research

STEP	POINTS TO CONSIDER
**Recruitment**<br>Recruitment of a PRP through the STAP-project (speed date; coordinator) using a written profile or via personal contacts.	• Preferably two PRPs<br>• Preferably people with the researched condition.
**Lay summary**<br>Writing a lay summary in the country's language of the research (in case a protocol or research application is already available).<br>** Writing a lay summary together might be a stimuli at the start of a (new) collaboration.*	• Involve PRPs as early as possible; Preferably from the very beginning of idea conception.<br>• Follow existing procedures for writing a lay summary.
**First acquaintance**<br>The researcher introduces himself and tells a bit about his own, personal background. Then, the PRP introduces himself. It is important that both the researcher as the PRP express their expectations with regard to the collaboration. Personal background and interests scan be shared as well.<br>It is allowed to mention personal interests, for instance "this research is part of my PhD" or "I am looking for a meaningful use of my time".	• Think about the location.<br>• It is a pitfall to talk directly about the content of the research. Therefore, dose the amount of information and adjust it to the knowledge and expectations of the PRP(s).<br>• Nurturing too high expectations is also a pitfall. It is realistic to assume that the added value of patient involvement in basic research is smaller than in clinical research and will only become visible after a certain period of time.
**Take a walk through the laboratory**<br>For PRP(s) the work of a researcher comes to live when he or she is allowed to take a look behind the scenes. The PRP can also meet fellow researchers and see that basic research takes place in a certain environment (context).	
**Support and education**<br>After getting acquainted, it is useful to draw up a plan or approach for collaboration together. Discuss for instance frequency of meetings, how to stay in contact and what the needs are for support and education. Make clear agreements and write them down.	Think about:<br>• Offering accessible background information.<br>• Exchange email addresses and phone numbers.<br>• Plan meetings at times that are feasible for all.<br>• Organise timely and generous reimbursement of expenses.

**Collaboration**<br>Involve PRP(s) regularly in the conduct of the research. Explain what you have been doing, ask for feedback and present the next research steps.<br>Ask yourself the following question: *Which input do I need from the PRP(s) and how can they contribute to the research activities?*	**Potential contributions**<br>• Testing the clinical relevance of a research question.<br>• Reviewing lay summaries and other patient-facing information such as informed consent forms.<br>• Advise on research design, for instance regarding the acceptability of the burden and risks for study participants; recruitment and retention strategy.<br>• Keeping in touch with patient organisations, e.g. a letter of endorsement (support raising funds).<br>• Assist with the dissemination of results, for example by contacting editors of patient journals or writing articles for a patient audience.
**Reporting**<br>Make reports of meetings. Particularly if the researcher explains difficult concepts, it is beneficial to write them down as well. Good reporting also makes it easier to visualize the contributions (added value) that PRPs bring to the table.	• Reports and summaries form a useful reference guide, and are also helpful when preparing future PRP(s).
**Acknowledgement**<br>PRPs are often involved in research as volunteers. It is therefore important to acknowledge their contribution to the project and to express appreciation for it. This is often forgotten, but is important for maintaining motivation.	Examples<br>• Provide feedback on how PRPs' input has affected a project.<br>• Invitation to a New Year's meeting or to a PhD ceremony (public defense).<br>• Facilitating participation in a symposium or congress.<br>• Providing information, an interesting publication or a voucher.<br>• Offering co-authorship if the conditions for doing so are met.
**Evaluation**<br>Agree at the start when and how the collaboration will be evaluated. It makes sense to reflect together at regular times on the agreements made and whether they still apply (frequency of meetings, way of communicating, etc.)	

Legend: PhD (Doctoral study); PRP (patient research partner); STAP ('Key To Active Participation').
